# Multi-Scale Mechanical Characterization of Additively Manufactured GRCop-42 and GRCop-84 Alloys

**DOI:** 10.1007/s11837-025-07787-0

**Published:** 2025-10-03

**Authors:** MohammadBagher Mahtabi, Mojtaba Roshan, Gabriel Demeneghi, Paul Gradl, Wiktor Bednarczyk, Grzegorz Cios, Meysam Haghshenas

**Affiliations:** 1 Department of Mechanical, Industrial, and Manufacturing Engineering (MIME), University of Toledo, Toledo, OH 43606 USA; 2 NASA, Marshall Space Flight Center, Huntsville, AL 35808 USA; 3 Faculty of Metals and Industrial Computer Science, AGH University of Krakow, al. A. Mickiewicza 30, 30-059 Krakow, Poland; 4 Academic Centre for Materials and Nanotechnology, AGH University of Krakow, al. A. Mickiewicza 30, 30-059 Krakow, Poland

## Abstract

Additive manufacturing (AM) of copper alloys is gaining traction in high-performance applications such as rocket engine combustion chambers and heat exchangers. This study investigates the microstructural and mechanical behavior of two Cu-Cr-Nb alloys, GRCop-42 and GRCop-84, fabricated via laser powder bed fusion (L-PBF) and laser powder-directed energy deposition (LP-DED). A multiscale approach was used to relate micro/nanoscale features to macro-scale mechanical properties, with particular focus on the relationship between hardness and tensile strength. Indentation-derived properties, including plasticity index and elastic recovery, were also assessed. The results show that L-PBF-processed GRCop-42 specimens exhibit higher strength but lower elastic modulus compared to LP-DED counterparts. The strength–hardness correlation varied significantly depending on the processing method. Notably, for L-PBF samples with weak to moderate texture, hardness correlated well with tensile strength, with deviations between 2% and 16%. However, in LP-DED specimens exhibiting strong crystallographic texture, this correlation weakened due to anisotropic mechanical response. These findings highlight the influence of processing-induced microstructures on mechanical performance and provide practical insights into using hardness as a predictive metric for tensile properties in AM copper alloys.

## Introduction

Laser powder bed fusion (L-PBF) and laser powder-directed energy deposition (LP-DED) are metal additive manufacturing (AM) techniques, widely adopted for their ability to produce near-net-shape components with high complexity. Both processes build components layer by layer, offering significant design freedom, such as the ability to create intricate internal features, lightweight lattice structures, and complex geometries, while also reducing material waste and shortening fabrication cycles, making them valuable across various industries.^1^ However, they differ significantly in powder delivery, energy input mechanisms, and thermal histories, which influence the resulting microstructures and mechanical properties of the final parts.^2–4^ In the L-PBF process, each layer is formed by evenly spreading a thin powder layer over the build platform, followed by selective melting and fusion of the powder to the underlying solidified layer. L-PBF is well-suited for industries like aerospace and medical manufacturing due to its ability to create intricate parts with high dimensional accuracy by selectively melting thin layers of metal powder within an enclosed powder bed.^5^ In contrast, LP-DED employs a focused laser to create a melt pool while metal powder is blown through a nozzle into the melt pool, forming a deposited bead and ultimately building the part through a continuous powder-feeding process. LP-DED is ideal for the fabrication or repair of larger components, such as those in the aerospace, energy, and heavy machinery sectors.^6,7^ LP-DED offers a faster deposition rate, five times that of L-PBF (25 cm^3^/h vs. 5 cm^3^/h). This increased speed comes at the cost of reduced dimensional accuracy in the final parts.^8^ Together, these two AM techniques offer the potential of AM to replace or augment traditional manufacturing techniques across various sectors.

Copper-based alloys, with their outstanding thermal and electrical conductivity, combined with the ability of AM to create complex, functionally optimized structures, make them strong candidates for applications in extreme operating environments.^9,10^ Among the materials used in advanced AM techniques are the GRCop alloys (Cu-Cr-Nb), which were specifically designed to withstand the high thermal and mechanical loads within aerospace applications, particularly in rocket engine combustion chambers and heat exchangers.^11^ The Glenn research copper (GRCop) family, including GRCop-42 (Cu-4Cr-2Nb at%) and GRCop-84 (Cu-8Cr-4Nb at%), was developed by NASA's Glenn Research Center for use in rocket engine combustion chambers, where materials are required to withstand high heat flux conditions.^12^ One of the applications of GRCop alloys is in chamber liners, where components are subjected to severe plastic deformation caused by repeated thermal cycling and low-cycle fatigue conditions.^13,14^ In such environments, substantial thermomechanical stresses lead to strain accumulation, making it essential to assess the material’s plastic behavior under such service conditions. These copper alloys combine the high thermal and electrical conductivity of copper with dispersion strengthening through the incorporation of chromium and niobium, forming the Cr_2_Nb phase.^9,11^ This phase strengthens the alloy and enhances its oxidation resistance, making these materials ideal for high-temperature environments up to 800 °C. Several studies have examined the microstructure and mechanical properties of GRCop fabricated by AM techniques.^12,15,16^ The literature on L-PBF and LP-DED fabrication processes reveals key differences in microstructure, mechanical properties, and failure mechanisms.^3,13^

GRCop-42 was developed with a reduced Cr_2_Nb content to boost thermal conductivity at the expense of some mechanical strength. Its thermal conductivity is about 15% higher than that of GRCop-84, making it a suitable choice for channel-cooled rocket engine combustion chambers on launch vehicles.^16–18^ GRCop-84 offers excellent microstructural stability, thermal conductivity, creep resistance, tensile strength, and low-cycle fatigue life, optimized for use between 500 and 800 °C. It also exhibits lower thermal expansion than pure copper and dilute copper alloys up to 1000 °C.^19^ These properties make it a strong candidate for high heat flux applications like rocket combustion chambers, welding electrodes, fusion reactors, metal casting molds, and high-temperature heat exchangers.^20,21^ Both alloys have been widely adopted by NASA for high heat flux systems, especially where repeated thermal cycling is involved. While AM has enabled the creation of complex geometries, including channels in combustion chamber liners, techniques like L-PBF allow for the accurate fabrication of these designs. Structural reinforcement is then achieved by integrating L-PBF-produced copper liners with a secondary high-strength-to-weight alloy applied through LP-DED.^11^ By integrating these two AM techniques, GRCop alloys offer an effective solution for high-performance engines, balancing thermal conductivity and mechanical strength without the need for traditional fuel film cooling systems.

Comprehensive characterization of the mechanical properties of a material is essential for designing components, with tensile strength and hardness measurements being widely used for this purpose. Conducting tensile tests is also more challenging than hardness testing, as they must meet specific standards in terms of geometry and loading conditions, while hardness testing requires only a small block of the material and is faster to conduct.^22^ Several research efforts have been dedicated to identifying the correlation factors and associated parameters between hardness and tensile strength across a wide range of materials, and this correlation is well established for conventionally manufactured materials.^23–25^ This relationship is practically useful as it allows for the estimation of tensile properties, including yield stress (YS), through hardness measurements, providing a non-destructive and efficient method for evaluating material strength and reducing reliance on extensive tensile testing during the early stages of assessment. However, while this relationship is well documented in traditional materials, it remains insufficiently supported by statistical data for AM materials. The unique microstructure of AM materials, arising from complex processing conditions, complicates the direct application of these correlation factors. Thus, characterizing the relationship between hardness and tensile properties in AM materials can contribute to developing a method to predict mechanical properties and support AM material evaluation. Nanoindentation, with its load and depth-sensing capabilities, is a valuable tool for evaluating the local mechanical properties of materials with complex microstructures, such as GRCop alloys. By analyzing load–displacement data, this technique enables the determination of hardness and estimation of tensile properties, including elastic modulus, YS, and ultimate tensile strength (UTS), while requiring only small sample volumes, making it a cost-effective alternative to conventional tensile testing. The ability to measure load–displacement curves at nanoscales has made nanoindentation an important technique for studying heterogeneous microstructures and understanding the deformation mechanisms within them.

**Fig. 1:**
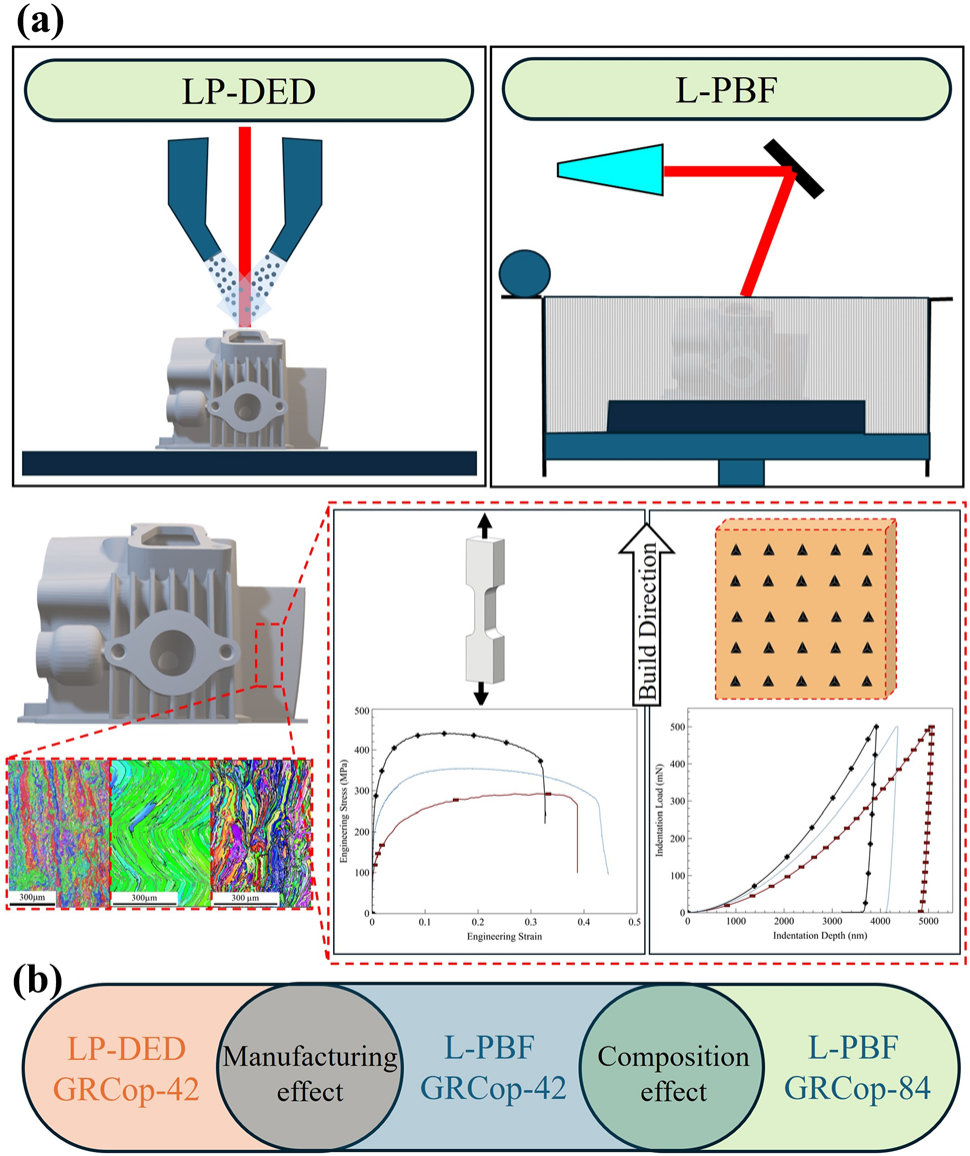
(a) Methodology overview for linking strength and hardness of GRCop alloys fabricated using two AM techniques, involving microstructural characterization (EBSD) and mechanical testing (hardness and tensile tests). (b) Schematic of the comparative approach used to assess the effects of alloy composition (L-PBF GRCop-42 vs. GRCop-84) and manufacturing technique (L-PBF vs. LP-DED for GRCop-42).

As a result, nanoindentation offers a practical means for the preliminary assessment of plastic deformation behavior in such alloys. By probing localized regions of interest, this technique provides valuable insights into the material’s response under plastic deformation, particularly in cases where conventional mechanical testing is constrained by sample size or geometry. By establishing empirical relationships between nanoindentation hardness and tensile properties, this study aims to create a statistical hardness–strength relationship for GRCop-42 fabricated using L-PBF and LP-DED techniques, as well as L-PBF GRCop-84. This hardness–strength relationship will serve as a fast and efficient method to estimate the strength of GRCop alloys when destructive testing is impractical, especially for novel materials and small-scale samples. Figure 1a shows the workflow of the methodology used to establish the hardness–strength relationship, which involves microstructural characterization and mechanical testing of GRCop-42 and GRCop-84 fabricated using L-PBF and LP-DED techniques. Although the primary focus of this study is on establishing the strength–hardness correlation within the GRCop family of alloys fabricated using two AM techniques (i.e., L-PBF and LP-DED), additional supportive comparisons have been performed throughout the paper to link microstructural features to mechanical properties and to aid in interpreting the observed mechanical behavior. These comparisons were conducted systematically, as the selected materials and manufacturing techniques share overlapping characteristics that allow the effects of alloy composition and manufacturing technique to be examined independently. To assess the effect of alloy composition, the AM process (i.e., L-PBF) was held constant while comparing the properties of L-PBF GRCop-42 and L-PBF GRCop-84. Conversely, to explore the influence of manufacturing technique, the alloy (GRCop-42) was kept constant while comparing samples produced using L-PBF and LP-DED. Figure 1b illustrates how these comparisons among the selected alloys help isolate and reveal the effects of both composition and manufacturing method. Additionally, nanoindentation-derived parameters, including plasticity index, elastic recovery, and wear response, were evaluated to provide further insights into the material’s elastic–plastic behavior and to enable a comprehensive assessment of its mechanical performance.

## Experimental

### Materials and Sample Production

Two AM techniques, L-PBF and LP-DED, have been employed to fabricate three classes of specimens: L-PBF GRCop-42, L-PBF GRCop-84, and LP-DED GRCop-42. This classification also allows for a systematic evaluation of how manufacturing techniques and alloy compositions influence material performance in these two powder-based AM processes. Powders were sourced from various lots, with each vendor applying distinct processing methods. All powders met the specifications for chemical composition and particle size distribution, as defined by Gradl et al.^11^

**Fig. 2:**
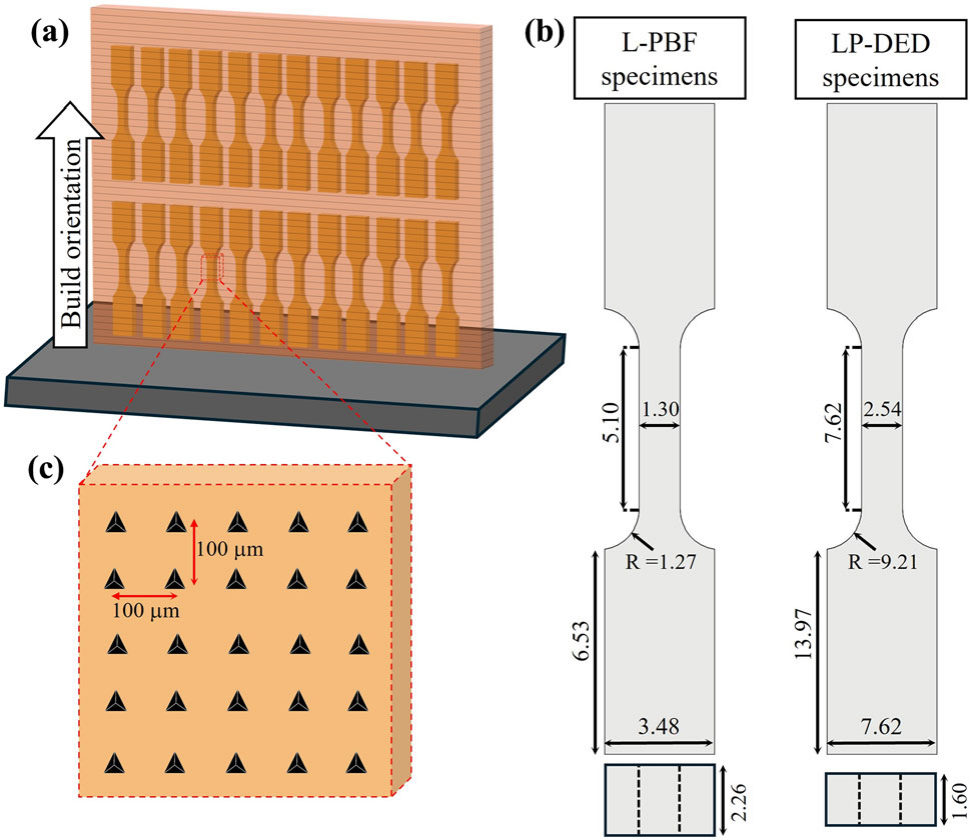
Schematic showing (a) the positions from which tensile and nanoindentation specimens were taken from the original build section, (b) the geometry and dimensions of the tensile specimens (dimensions in mm), and (c) the nanoindentation and microstructure sample, indicating how the sample was extracted from the specimen.

Figure 2a illustrates how the tensile and nanoindentation specimens were sectioned from the original build section. For the L-PBF GRCop-42 class, eight groups (Groups 1–8) were fabricated, with each group consisting of 12 samples. For the L-PBF GRCop-84 class, two groups (Groups 9 and 10) were produced, also with 12 samples per group. The builds for both GRCop-42 and GRCop-84 were manufactured by different vendors, employing their proprietary processing methods. The vendors were instructed to use best practices for manufacturing GRCop-42 and − 84. Further parameter details on the fabrication process are available in Refs. 26 and 27. For the LP-DED GRCop-42 class, two groups of samples (Groups 11 and 12) were fabricated, each consisting of four specimens. These samples were produced using powders with slight variations in Cr/Nb ratios using an RPMI 222 machine equipped with an infrared laser and a back-and-forth (striping) deposition strategy.^26,28^ To reduce manufacturing-induced variability, particularly associated with build orientation, all specimens were fabricated in the upward direction.^29^

### Microstructural and Mechanical Characterization

For microstructural characterization, samples were hot-resin-mounted in an orientation parallel to the build direction. The samples were extracted from the location corresponding to the gauge section of the tensile specimens. Polishing was carried out to achieve a mirror-like finish, starting with coarse grit and progressing to a 7000-grit abrasive, followed by final polishing using 0.3-µm and 0.05-µm alumina suspensions. For detailed microstructural analyses, electron backscatter diffraction (EBSD; NordlysMax3; OXFORD Instruments) was employed with a step size of 2.0 µm. For EBSD, the samples were further electropolished using a Struers Lectropol-5 at 6 V for 15 s. The electropolishing solution comprised 100 mL of deionized water, 78 mL of phosphoric acid, and 21 mL of sulfuric acid.

Uniaxial tensile testing was carried out on dog-bone specimens according to ASTM E8 standard^30^ at room temperature using a servo-hydraulic materials testing system equipped with a 5-kN load cell and an Epsilon extensometer to measure displacement. The test was initiated with a strain rate of 0.015 s^−1^ to identify the 0.2% offset YS, then continued at a secondary rate of 0.01 in/min until sample failure. A digital caliper was used to determine the cross-sectional area in this study, as all the specimens were machined to achieve a uniform surface finish and minimize the influence of surface texture, particularly roughness. Width and thickness measurements were taken at three random locations along the gauge section, averaged, and used to calculate the cross-sectional area. Figure 2b illustrates the geometry and dimensions of the tensile specimens.

A nanoindentation platform (iMicro; KLA Instruments) was employed for small-scale mechanical testing. One sample from each group was selected for the nanoindentation tests and extracted from the same area as the tensile specimen gauge section. A systematic grid pattern was applied to determine indentation points along the build direction, with uniform spacing between each indent following ISO 14577.^31^ A Berkovich indenter with a 100-nm tip radius was used for testing, performing 25 indents per sample at a maximum load of 500 mN and a loading/unloading rate of 25 mN/s. Nanoindentation load–depth curves were recorded after a 2-s hold at peak load. The indents were arranged in a systematic 5 × 5 grid pattern over a 400 × 400 µm^2^ area, with a spacing of 100 µm between each indent to minimize grain-size-induced variability and ensure statistical reliability of the measured mechanical properties (Fig. 2c).

## Results and Discussion

### Microstructure

**Fig. 3:**
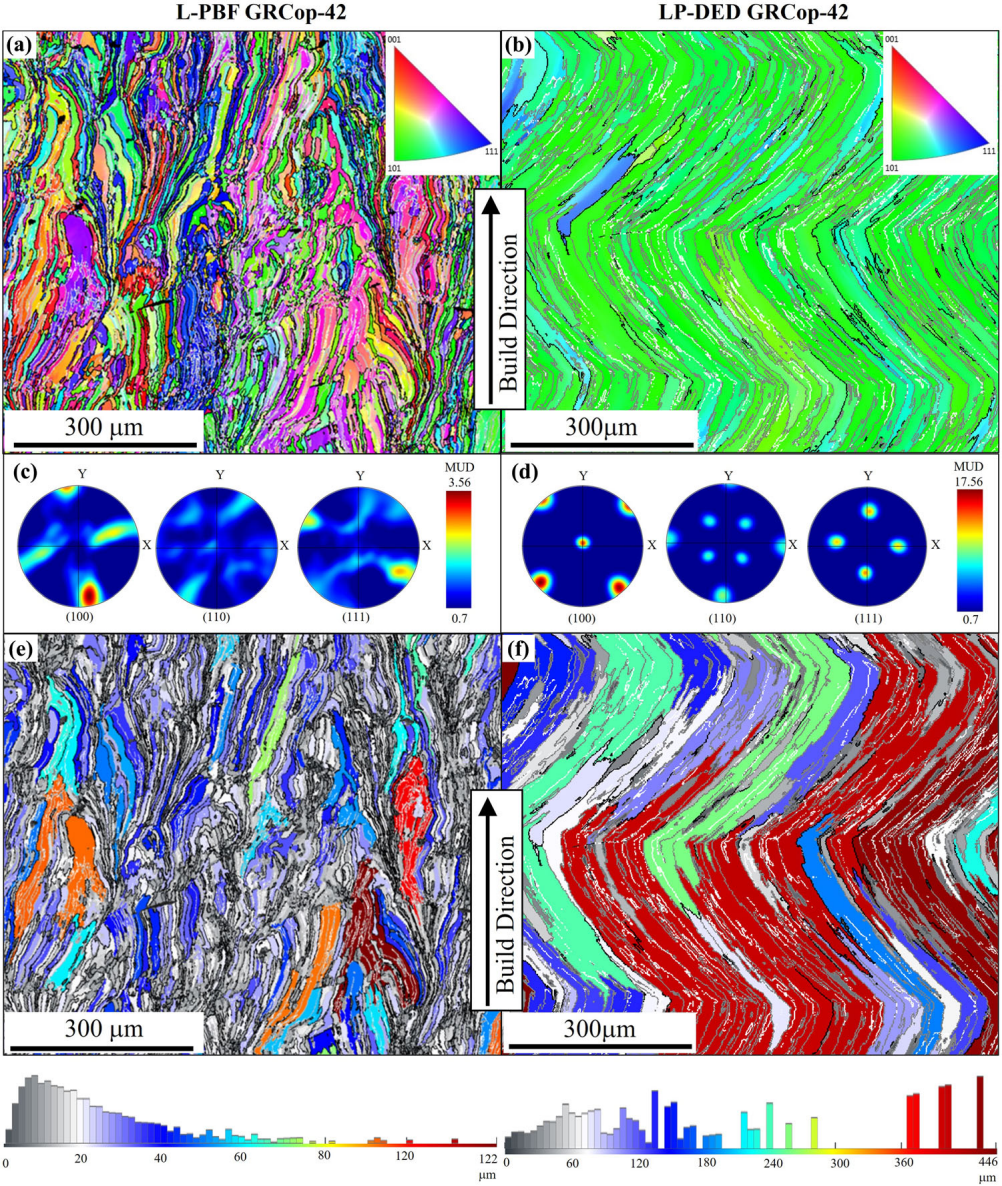
EBSD of GRCop-42 fabricated by L-PBF and LP-DED: (a, b) IPF maps in the plane parallel to the build direction show distinct grain structures: L-PBF (a) exhibits equiaxed grains with a scattered color distribution, indicating no strong visual grain orientation; LP-DED (b) shows columnar grains with a dominant [101] orientation, indicating strong texture. (c, d) PFs (in MUD units) show moderate texture (001) in L-PBF (c) and a much stronger texture (001) in LP-DED (d), aligned with the laser scanning path. (e, f) Grain size maps reveal finer grains in L-PBF (e), mostly under 50 μm, and larger grains in LP-DED (f), reaching up to 446 μm.

EBSD analysis was performed on the plane parallel to the build direction. It should be noted that one sample is shown as a representative for each class. Figure 3 presents the EBSD maps for the GRCop-42 alloy fabricated using the L-PBF and LP-DED techniques. The EBSD reveals the epitaxial grain growth between the deposited layers, a result of re-melting previously deposited layers. This re-melting process encourages the formation of elongated grains aligned parallel to the build direction, primarily due to the higher heat conduction rate in the solidified layers compared to the virgin powder.^32^ As a result, directional solidification occurs within the melt pool, where most of the heat is released from the lower layers, generating a thermal gradient that drives the growth of columnar grains toward the melt pool center.^33^ Moreover, copper’s high thermal conductivity further accelerates heat dissipation, reinforcing the thermal gradient and promoting grain growth along the heat flow direction. Before sufficient cooling can trigger nucleation, the thermal gradient continues to guide the alignment of the grains.^34^ As can be seen in Fig. 3, distinct differences are observed between the two fabrication techniques: the L-PBF sample shows thinner but highly elongated grains, resulting in a higher average grain aspect ratio (~ 5.4 for L-PBF vs. ~ 4.2 for LP-DED), while the LP-DED sample exhibits continuous agglomeration of subgrains forming zig-zag bands composed of low-angle grain boundaries.

The inverse pole figure (IPF) map of L-PBF GRCop-42, shown in Fig. 3a, exhibits a microstructure with a scattered color distribution. This pattern suggests minimal preferential grain orientation, indicating an isotropic structure with uniform mechanical properties, although this observation is based on localized microscopic analysis. In contrast, the IPF map of LP-DED GRCop-42 shown in Fig. 3b reveals that the grains are predominantly oriented in the [101] direction, as indicated by the green color, suggesting that LP-DED GRCop-42 exhibits a preferred crystallographic orientation (texture). The pole figures (PF) are presented in multiples of uniform distribution (MUD), where a MUD of 1 represents a completely random distribution of grain orientations and higher values reflect strong crystallographic texture within the material. For L-PBF GRCop-42, the PF revealed a moderate crystallographic texture along the (001) plane with the MUD value of 3.56 (Fig. 3c), while LP-DED GRCop-42 exhibits a strong crystallographic texture along the (001) plane with a MUD value of 17.56. As can be seen in Fig. 3d, a well-defined crystallographic texture of the type (001) < 101 > was observed for the LP-DED GRCop-42 sample, where the (001) planes are oriented parallel to the build direction, and the < 101 > directions align with the laser scanning path. This phenomenon can be explained by the tendency of face-centered cubic^35^ crystals to preferentially grow along the < 001 > direction during solidification.^35,36^ Such directional growth typically aligns with the path of maximum thermal gradient, which in AM is generally perpendicular to the build plane and thus parallel to the build direction.^35,37^ The crystallographic texture in AM processes is influenced by factors such as the laser scanning strategy, geometric and morphological characteristics of the melt pool, and laser energy density.^38,39^ During L-PBF, texture development begins with the growth of grains from the substrate through epitaxial solidification, which forms cellular structures due to rapid cooling.^40^ The grain size distribution map for both LP-DED and L-PBF GRCop-42 is presented in Fig. 3e and f, respectively. While for L-PBF, GRCop-42 grain size distribution shows that the majority of the grains are smaller than 50 μm with a mean value of 22 μm (Fig. 3e), LP-DED GRCop-42 exhibits comparatively larger grains with a noticeable discrepancy in grain size distribution, where the equivalent grain diameter even reaches up to half a millimeter. Moreover, it should be noted that, due to the complexity and elongation of the grain structures observed in both L-PBF and LP-DED samples, the maximum Feret diameter was used for grain size quantification, as it better captures anisotropic features than the area-weighted equivalent circle diameter. To account for the high fraction of low-angle grain boundaries, especially in the LP-DED sample, a lower misorientation threshold of 5° was applied to define the grain boundaries, providing a more accurate representation of the subgrain structure.

**Fig. 4:**
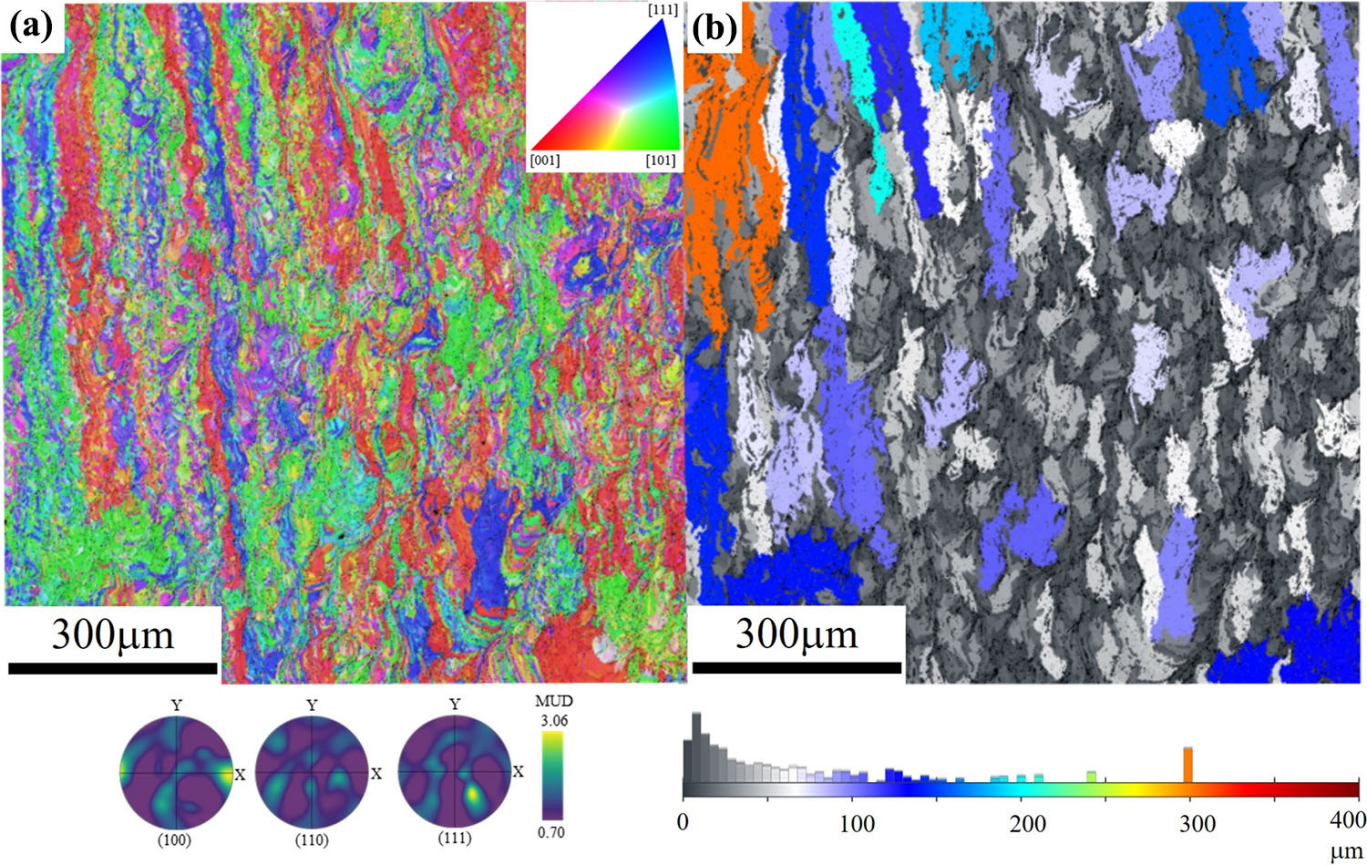
EBSD maps of L-PBF GRCop-84 from the plane parallel to the build direction: (a) IPF map shows a slight preference for $\left\langle {001} \right\rangle$ orientation along the build direction, with a maximum MUD value of 3.06, indicating weak crystallographic texture. (b) The grain map reveals elongated grains oriented along the build direction, with a more irregular and varied size distribution compared to L-PBF GRCop-42.

The EBSD results of GRCop-84 are presented in Fig. 4. The IPF map in Fig. 4a indicates that the sample exhibits a slight preference for the $\left\langle {001} \right\rangle$ texture along the build direction, with a maximum MUD value of 3.06. In comparison to L-PBF GRCop-42, which has a higher maximum MUD value of 3.56 (see Fig. 3a), GRCop-84 appears to be slightly less textured. Meanwhile, the grain map in Fig. 4b reveals an elongated microstructure primarily oriented along the build direction. Compared to L-PBF GRCop-42, the grain distribution appears more irregular. While differences in the overall nature of the manufacturing techniques (i.e., L-PBF vs. LP-DED) or composition (L-PBF GRCop-42 vs. L-PBF GRCop-84) were considered in the comparisons made in this study, other factors, such as sample location on the build plate,^41^ build height,^42^ and specific printing parameters,^43,44^ may still influence the resulting microstructure and mechanical properties (Table I).

**Table I:** Chemical composition of L-PBF and LP-DED GRCop-42 and GRCop-84 samples

	Build ID	Cu	Cr (wt.%)	Nb (wt.%)	Al (wt.%)	Si (wt.%)	Fe (wt.%)
L-PBF GRCop-42	1	Balance	3.29	2.95	0.050	0.022	0.007
	2	Balance	3.25	2.89	0.048	0.021	0.007
	3	Balance	3.31	3.08	0.048	0.020	0.004
	4	Balance	3.23	2.86	0.041	0.024	0.005
	5	Balance	3.22	2.82	0.042	0.039	0.009
	6	Balance	3.20	2.77	0.041	0.034	0.009
	7	Balance	3.16	2.75	0.026	0.011	0.003
	8	Balance	3.22	2.92	0.043	0.027	0.006
L-PBF GRCop-84	9	Balance	6.48	5.77	0.004	0.006	0.002
	10	Balance	6.52	5.61	0.023	0.031	0.004
LP-DED GRCop-42	11	Balance	3.13	2.9	0.040	0.032	0.004
	12	Balance	3.30	2.9	0.053	0.035	0.003

### Tensile Results

Tensile test results for all the specimens, including YS, UTS, modulus of elasticity, and strain-hardening exponent, are provided in Table II. As presented, the tensile properties of the studied materials exhibit notable differences. L-PBF GRCop-42 exhibits an average YS of 200 ± 6 MPa and a UTS of 348 ± 11 MPa. It has a strain-hardening exponent (n) of 0.198 ± 0.012 and an elastic modulus (E) of 99.6 ± 10.5 GPa. The elongation to failure averages 37.4 ± 7.9%. In contrast to L-PBF GRCop-42, LP-DED GRCop-42 exhibits a lower strength, with an average YS of 167 ± 1 MPa and UTS of 298 ± 1 MPa. However, it shows higher elongation to failure, strain-hardening exponent, and elastic modulus of 41.3 ± 3.6%, 0.294 ± 0.002, and 123.5 ± 9.2 GPa, respectively. L-PBF GRCop-84, on the other hand, shows higher strength but lower ductility compared to L-PBF GRCop-42, with an average YS of 256 ± 7 MPa, UTS of 444 ± 2 MPa, and elongation to failure of 34.4 ± 2.8%. Its elastic modulus and strain-hardening exponent are 111.5 ± 8.4 GPa and 0.198 ± 0.001, respectively.

**Table II:** Summary of the tensile results for L-PBF GRCop-42 and GRCop-84, as well as LP-DED GRCop-42

	Group ID	No. of specimens	YS (MPa)	UTS (MPa)	n	E (GPa)	Elongation (%)
L-PBF GRCop-42	S1	12	195 ± 6.89	356 ± 1.93	0.218	97.2 ± 4.00	42.17 ± 2.33
	S2	12	206 ± 12.89	342 ± 9.03	0.205	88.7 ± 7.99	42.15 ± 2.64
	S3	12	194 ± 12.68	342 ± 15.79	0.185	92.5 ± 13.10	40.64 ± 3.87
	S4	12	205 ± 10.4	361 ± 2.83	0.209	107.8 ± 5.93	36.63 ± 3.68
	S5	12	207 ± 10.07	330 ± 31.23	0.201	117.6 ± 16.20	19.83 ± 10.41
	S6	12	199 ± 7.86	357 ± 3.45	0.178	97.4 ± 17.17	41.25 ± 2.01
	S7	12	199 ± 14.13	347 ± 9.38	0.199	100.5 ± 18.82	39.46 ± 6.26
	S8	12	198 ± 7.58	348 ± 5.24	0.207	95.2 ± 17.99	42.06 ± 2.43
L-PBF GRCop-84	S9	12	261 ± 10.00	443 ± 9.03	0.197	117.4 ± 7.93	36.35 ± 2.58
	S10	12	251 ± 5.24	446 ± 5.38	0.199	105.6 ± 6.41	32.45 ± 4.35
LP-DED GRCop-42	S11	4	166 ± 3.84	299 ± 3.18	0.292	130.0 ± 8.21	38.76 ± 2.25
	S12	4	168 ± 5.36	298 ± 2.16	0.295	117.0 ± 7.81	43.89 ± 4.65

**Fig. 5:**
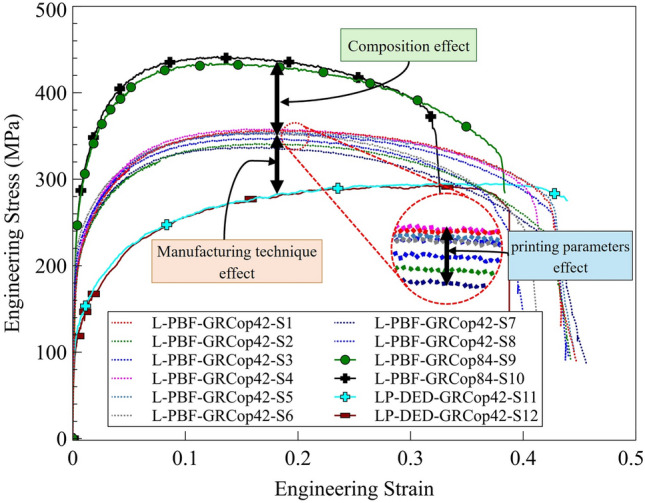
Engineering stress–strain curves of L-PBF GRCop-84, L-PBF GRCop-42, and LP-DED GRCop-42. When comparing the effect of composition, GRCop-84 exhibits higher strength than GRCop-42. When comparing the effect of manufacturing method for GRCop-42, the L-PBF process results in higher strength and lower ductility than LP-DED.

The tensile test results for L-PBF GRCop-42, L-PBF GRCop-84, and LP-DED GRCop-42 specimens are summarized in Fig. 5, where one representative curve is shown for each group. L-PBF GRCop-42, with its finer grain structure, exhibited higher strength compared to LP-DED GRCop-42. A finer microstructure enhances tensile strength by creating more grain boundaries, which act as barriers to dislocation movement. This resistance to dislocation motion requires more energy to deform the material, thereby increasing its tensile strength.^45^ In contrast, LP-DED GRCop-42, with its strong crystallographic texture, showed lower tensile strength. When the grains are strongly textured, mechanical performance can become directionally dependent, enhancing properties along certain orientations while diminishing them along others. This anisotropic behavior means that, if the loading axis does not align with the dominant grain orientation, the material may exhibit reduced strength.^46^ This highlights the anisotropic nature of texture-driven microstructures, where mechanical properties can vary significantly depending on the loading direction relative to the grain orientation. In the case of LP-DED GRCop-42, the strong texture may have contributed to increased elastic stiffness along certain directions, yet reduced strength under uniaxial tensile loading if that loading direction did not align with the favorable crystallographic orientations. Such anisotropic effects are more pronounced in materials like copper, where elastic and plastic responses are highly sensitive to crystallographic direction. On the other hand, grain size plays a key role in determining the mechanical properties of metals.^47,48^ Therefore, the differences in tensile properties of the alloy might also be linked to variations in grain size distribution. LP-DED GRCop-42 exhibited significantly larger average grain sizes compared to its L-PBF counterpart. According to the Hall–Petch relationship, smaller grains lead to higher strength, while coarser grains typically result in lower strength but higher ductility.^49,50^ As a result, the larger grain size in LP-DED GRCop-42 contributed to its lower UTS and greater elongation to failure compared to the L-PBF process. While the influence of specific process parameters and machine-related factors (such as build volume and thermal gradients) could cause some variation within each technique, the differences in mechanical properties between the two AM techniques (L-PBF vs. LP-DED) were more prominent than the variations observed within each technique.

**Fig. 6:**
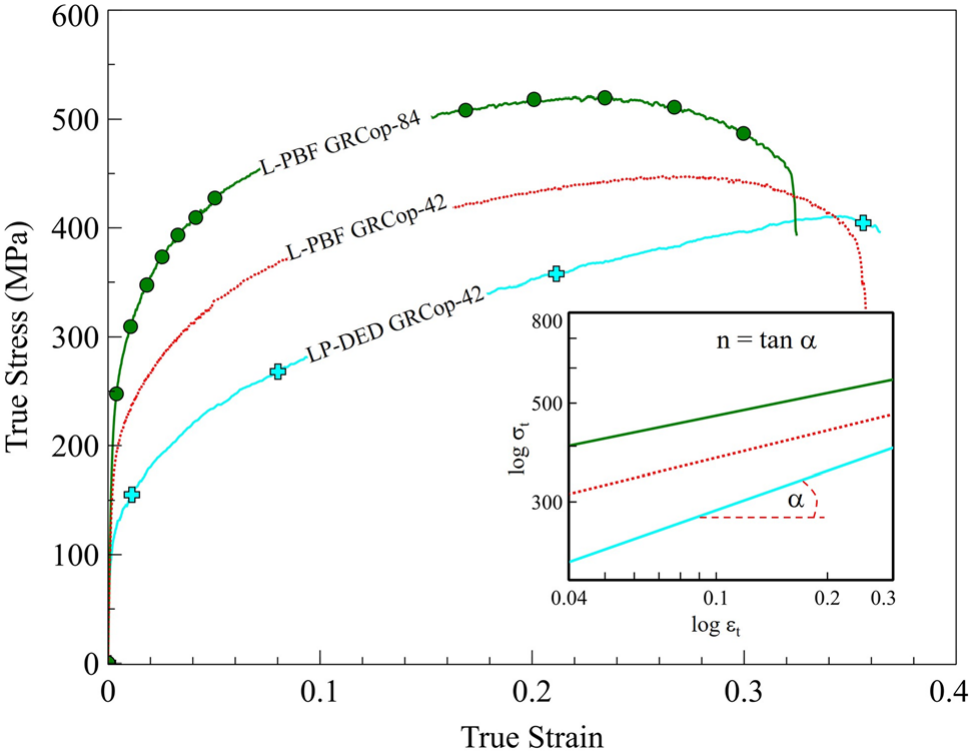
True stress–strain curves and corresponding log–log plots for representative specimens: L-PBF GRCop-42 (S1), L-PBF GRCop-84 (S9), and LP-DED GRCop-42 (S11). When comparing the effect of composition, L-PBF GRCop-42 and GRCop-84 show similar strain-hardening exponents. In contrast, when comparing manufacturing methods for GRCop-42, LP-DED GRCop-42 exhibits a higher exponent.

Regardless of the manufacturing technique, the tensile results reveal the work-hardening behavior of the GRCop alloys, which occurs when the material becomes stronger as it undergoes plastic deformation, with dislocations accumulating and interacting to resist further deformation. This process is described by the Hollomon equation:^51^

1 $${\sigma }_{t}=K\times {\varepsilon }_{t}^{n}$$

where *σ*_t_ represents the true stress, *K* is the strength coefficient, *ε*_*t*_ denotes the true strain, and *n* is the strain-hardening exponent. The work-hardening behavior of the materials was analyzed by plotting the true stress versus true strain curve in a log–log scale, where the slope of the line corresponds to the strain-hardening exponent, and the intercept represents the strength coefficient (*K*), as shown in Fig. 6. The true stress–true strain curves for three representative specimens from each class, shown in Fig. 6, reveal that L-PBF GRCop-42 and GRCop-84 exhibit comparable strain-hardening exponents (~ 0.2), while LP-DED GRCop-42 demonstrates a significantly higher value (~ 0.29).

The differences in mechanical properties observed between the L-PBF and LP-DED specimens can be traced back to the distinct thermal histories, cooling rates, and laser sources used in each process.^52,53^ In L-PBF, the rapid heating and cooling cycles result in finer microstructures, enhancing strength but reducing ductility. Conversely, LP-DED involves slower cooling and more uniform heat distribution, leading to larger grains and potentially lower strength but improved ductility.^2^

### Nanoindentation-Driven Data

**Fig. 7:**
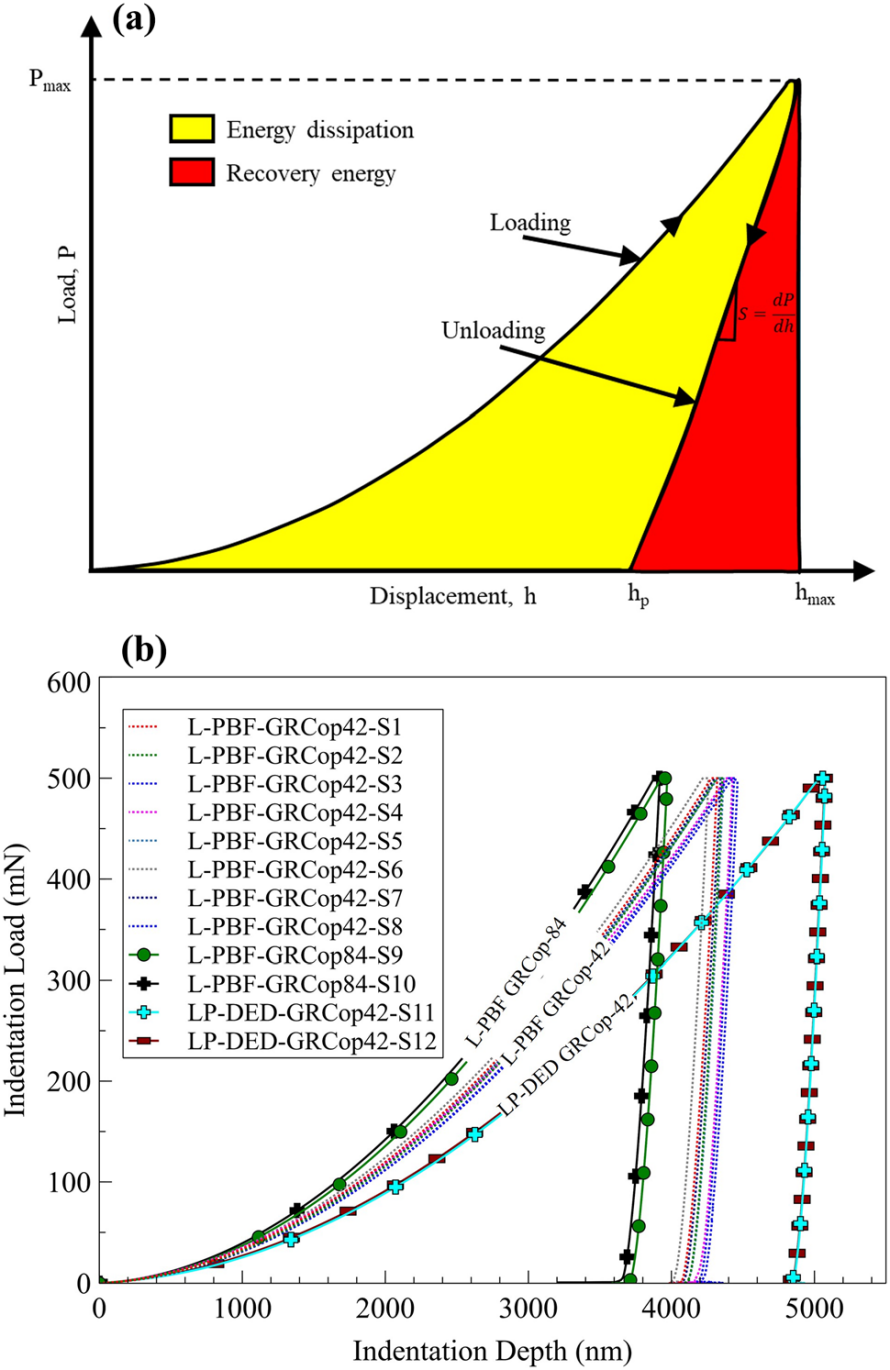
(a) Schematic of the load–displacement curve during nanoindentation, including loading, holding, and unloading sequences. (b) Average indentation P–h curves for L-PBF GRCop-42, GRCop-84, and LP-DED GRCop-42, showing that L-PBF GRCop-84 has a lower indentation depth (higher hardness) than L-PBF GRCop-42, while LP-DED GRCop-42 exhibits a higher indentation depth (lower hardness).

During indentation testing, following the complete loading and unloading cycle, key parameters such as load (*P*), depth (*h*), and time are recorded. These data aid significantly in evaluating the mechanical properties of materials, such as hardness, reduced modulus, wear resistance index, and plasticity index. Figure 7a schematically presents the process of loading and unloading steps, illustrating the characteristic load–depth curve. From this curve, additional insights such as energy dissipation during deformation and recovery energy, which reflects the material's elastic behavior, can also be derived. Indentation load–depth results of L-PBF GRCop-42 and GRCop-84, as well as LP-DED GRCop-42 samples, are shown in Fig. 7b. An analysis of the indentation load curve provides insight into the material’s strength under constant loading (i.e., softer material will exhibit higher indentation depth). Accordingly, the L-PBF GRCop-84 showed lower indentation depth among all samples, indicating higher hardness. On the other hand, the higher indentation depth of LP-DED GRCop-42 specimens indicates that these samples are softer than the L-PBF GRCop-42 and GRCop-84. While the L-PBF GRCop-42 measured indentation depth reveals a hardness value between the samples. Although localized microstructural features like Cr_2_Nb precipitates may influence individual indents, the high indentation load (500 mN) likely averages over multiple microstructural features, contributing to the consistent trends observed within each group.

The higher indentation depth observed in the nanoindentation tests for LP-DED GRCop-42 samples suggests that these specimens exhibit softer behavior at the micro/nanoscale, despite their higher elastic modulus. The deeper indentation may be attributed to the anisotropic microstructure of the LP-DED GRCop-42 material, where the highly textured grain structure likely contributes to its lower resistance to plastic deformation.^54,55^ The directional solidification in LP-DED leads to a more pronounced grain alignment, which may influence how the material deforms under local loading. In contrast, the L-PBF GRCop-42 samples, which have a finer microstructure with moderate texture, exhibit less indentation depth, indicating greater resistance to localized plastic deformation. Therefore, L-PBF samples may result in higher hardness and less plasticity under indentation, despite their lower elastic modulus.

This behavior suggests that while LP-DED GRCop-42 is stiffer in terms of elastic deformation, it shows greater susceptibility to plastic deformation at the micro/nanoscale, highlighting the complex relationship between indentation depth and microstructural features, such as grain texture. This observation is notable because, while it is generally known that harder materials tend to have higher elastic and shear moduli, the relationship between hardness and these moduli is not always linear.^56^ While hardness does not have a direct relationship with shear or bulk moduli, it can offer an approximate indication of a material’s elastic behavior. Labonte et al.^57^ described indentation hardness as a composite property that captures resistance to both elastic and plastic deformation. Furthermore, changes in the indentation modulus can influence the measured hardness. True hardness (theoretical hardness) primarily reflects plasticity, while Young’s modulus is a measure of elasticity.^58^

### Strength–Hardness Correlation

Predicting tensile strength through nanoindentation is challenging due to the inherent differences between indentation and tensile testing methods. Tensile testing applies uniaxial stress, while nanoindentation generates a complex stress field that depends on the geometry of the indenter, such as the Berkovich tip.^59^ Additionally, nanoindentation typically induces a plastic strain of around 7%, whereas YS in tensile testing is defined at the onset of plastic deformation, usually at 0.2% strain.^60^ These disparities complicate the direct conversion of nanoindentation hardness data into tensile properties. Various models, ranging from simple empirical relationships like the Tabor equation^61^ to more complex analytical approaches involving parameters such as the strain-hardening coefficient,^61,62^ have been developed to bridge this gap. For conventional polycrystalline materials, empirical models are commonly used to relate hardness to tensile properties, often expressed as:

2 $$H={C}_{s}\times {\sigma }_{s}$$

where *σ*_*s*_ represents the uniaxial flow strength (either YS or UTS), *H* denotes hardness, and *C*_*s*_ is a material constant. For metals with minimal strain hardening, *C*_*s*_ is typically between 3 and 3.45, for predicting YS (C_YS_) and UTS (C_UTS_), respectively.^25,63^ The flow strength in this context corresponds to the level of plastic strain associated with the hardness test, which is largely determined by the geometry of the indenter tip.

Additionally, these relationships, initially developed for coarse-grained polycrystalline materials, are applicable only under ideal plastic response conditions, where local plastic deformation during indentation does not affect the material's strength. For materials that exhibit significant work hardening, a correction factor is necessary.^25^ It has been shown that the work-hardening behavior of fine-grained materials produced through severe plastic deformation is markedly different from that of unprocessed coarse-grained metals and alloys.^64^ Therefore, applying these constraint factors to the engineering design of components, particularly those composed of fine-grained or ultrafine-grained materials, may result in substantial overestimation or underestimation of their strength. Cahoon et al.^65^ proposed expressions that relate hardness to YS and UTS while accounting for the effects of work hardening, as shown in Eqs. 3 and 4. In these equations, *H* is the Vickers hardness in MPa, and *n* represents the strain-hardening exponent. It should be noted that applying these equations requires prior knowledge of the strain-hardening exponent, which can be directly obtained through a uniaxial tensile test or estimated indirectly using empirical relationships reported in the literature:^66^

3 $${\sigma }_{UTS}=(\frac{H}{{C}_{s}}){(\frac{n}{0.217})}^{n}$$

4 $${\sigma }_{y}=(\frac{H}{{C}_{s}}){(0.1)}^{n}$$

By applying Eqs. 3 and 4 in conjunction with the tensile test results by considering the work-hardening exponent, the values of C_s_ for the YS and UTS (i.e., C_YS_ and C_UTS_, respectively) of the GRCop alloys in this study can be determined (see Table III). The comparison reveals that, while the majority of the calculated correlation factors for UTS (C_UTS_) fall within the range proposed by Cahoon, notable variations are observed in the correlation factors across different alloys. Notably, Tabor^61^ suggested that C_s_ values typically fall between 2.9 and 3, while Cahoon et al.^65^ proposed a slightly broader range of 2.9–3.1. However, for L-PBF GRCop-42 in this study, the observed range is broader, with correlation factors between 2.93 and 3.25. This deviation may be attributed to variations in powder sources from different vendors and the differences in manufacturing parameters. In contrast, the correlation factors for L-PBF GRCop-84 (2.92–2.99) and LP-DED GRCop-42 (2.91–2.93) are notably lower and more constrained. These variations could be attributed to differences in microstructural features, such as grain size, phase distribution, and porosity, which are inherently affected by the processing methods used for each material. It should be noted that, while porosity is a critical factor in characterizing AM materials, given its significant influence on mechanical performance, direct porosity analysis was not conducted in this study due to limited access to non-destructive evaluation equipment. Additionally, the fabrication parameters were optimized by the manufacturer to achieve maximum density and minimize defects. Moreover, among mechanical properties, porosity tends to have a more pronounced effect on fatigue behavior than on tensile loading, as pores act as stress concentrators under cyclic conditions. In contrast, during tensile testing, the axial load is more uniformly distributed across the specimen’s cross-section, and, among tensile properties, elongation at fracture is typically more sensitive to porosity than tensile strength.^67,68^ Such differences in correlation factors emphasize the need to tailor predictive models to account for the unique properties of each alloy and manufacturing technique, ensuring a more accurate representation of mechanical performance.

**Table III:** Hardness–strength correlation factors considering work-hardening plastic for L-PBF GRCop-42 and GRCop-84, as well as LP-DED GRCop-42

Material	Manufacturing technique	Group ID	HV (MPa)	C_YS_	C_UTS_
GRCop-42	L-PBF	S1	1109	3.45	3.12
		S2	1090	3.31	3.15
		S3	1053	3.55	2.99
		S4	1065	3.21	2.93
		S5	1088	3.31	3.25
		S6	1143	3.81	3.09
		S7	1096	3.49	3.11
		S8	1037	3.25	2.95
GRCop-84	L-PBF	S9	1319	3.22	2.92
		S10	1356	3.42	2.99
GRCop-42	LP-DED	S11	797	2.45	2.91
		S12	798	2.41	2.93

Among the most notable properties derived from indentation data are the reduced modulus (*E*_*r*_) and the indentation hardness (*H*_*ind*_). The reduced modulus can be obtained using the maximum load (*P*_*max*_), maximum depth (*h*_*max*_), and the slope of the unloading curve (see Fig. 7a). The widely adopted Oliver–Pharr method^69^ defines the relationship for indentation hardness (*H*_*ind*_) as:

5 $${H}_{ind}= \frac{{P}_{max}}{{A}_{c}}$$

where *A*_*c*_ represents the projected contact area of the indentation. For a Berkovich indenter, the projected area is calculated using:

6 $${A}_{c}= 24.56 {h}_{p}^{2}$$

where *h*_*p*_ refers to the plastic depth, which is determined by:

7 $${h}_{p}= {h}_{max}- \varepsilon \frac{{P}_{max}}{S}$$

where *h*_*max*_ is the maximum penetration depth, *S* is the stiffness derived from the slope of the upper portion of the unloading curve, and *ε* is a constant specific to the indenter geometry, with a value of 0.75 for the Berkovich indenter. The value of *S*, the stiffness, can also be calculated from:

8 $$S=\frac{dP}{dh}=2\beta \sqrt{\frac{{A}_{c}}{\pi }} {E}_{r}$$

where *β* is a geometrical parameter associated with the indenter, with a value of 1.168 for the Berkovich indenter, and *E*_*r*_ is the reduced modulus (also called indentation modulus). The indentation tests provide a reduced modulus, which is the function of four parameters, including Poisson's ratios (ν_s_ and ν_i_) and elastic moduli (E_s_ and E_i_) of the indenter and the indented material, respectively, and can be calculated using:

9 $$\frac{1}{{E}_{r}}= {(\frac{1-{\nu }_{s}^{2}}{{E}_{s}})}_{sample}+{(\frac{1-{\nu }_{i}^{2}}{{E}_{i}})}_{indenter}$$

**Fig. 8:**
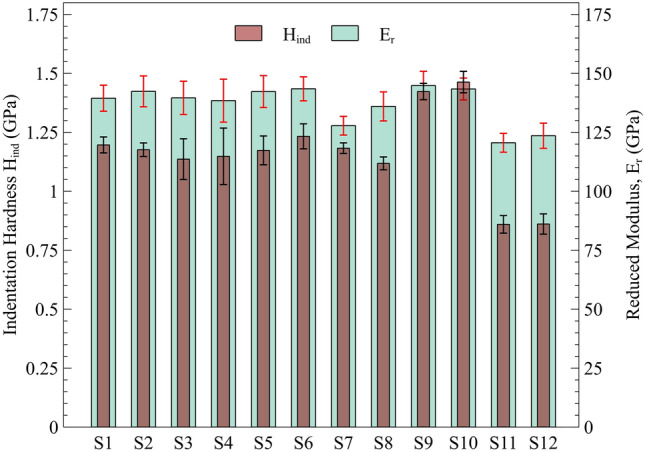
Indentation-derived hardness (H_ind_) and reduced modulus (E_r_) for GRCop samples: S1–S8 represent L-PBF GRCop-42, S9 and S10 represent L-PBF GRCop-84, and S11 and S12 represent LP-DED GRCop-42.

Fig. 8 summarizes the derived indentation hardness and reduced modulus of the materials studied. The results indicate that the L-PBF GRCop-84 samples exhibit 20–30% higher hardness compared to L-PBF GRCop-42, highlighting the influence of alloy composition and aligning with the tensile results. This enhancement in mechanical properties is primarily due to a greater volume fraction of Cr_2_Nb strengthening precipitates in GRCop-84, resulting from its higher chromium and niobium content. Although both alloys form the Cr_2_Nb phase, the increased precipitate density in GRCop-84 leads to superior hardness and tensile strength, whereas the reduced precipitate content in GRCop-42 results in decreased hardness and tensile strength relative to its GRCop-84 counterpart.^9,16,18^ In contrast, the LP-DED GRCop-42 samples show approximately 30% lower hardness than their L-PBF counterparts, suggesting that the L-PBF process results in a higher hardness value, likely due to finer microstructural features. The significantly lower hardness of LP-DED GRCop-42 may be attributed to a coarser microstructure. Furthermore, the lower indentation hardness of LP-DED GRCop-42 directly affects the predicted YS values, as determined using the model proposed by Gao et al.,^70^ as discussed below.

**Fig. 9:**
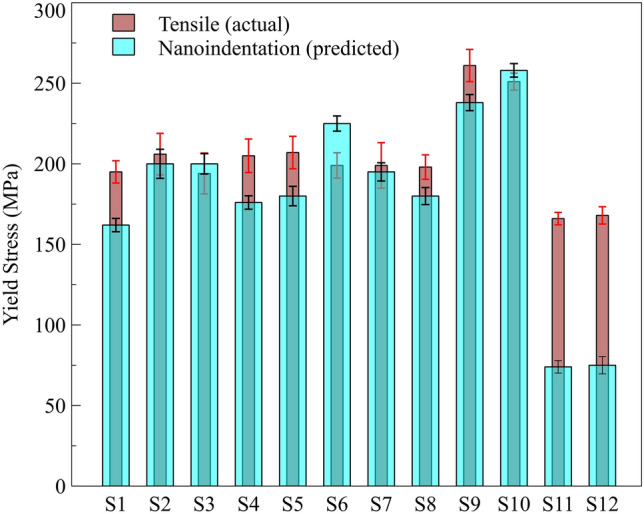
Comparison of yield stress predicted using nanoindentation with tensile test results for GRCop samples. *S1–S8* represent L-PBF GRCop-42, S9 and *S10* represents L-PBF GRCop-84, and *S11* and *S12* represent LP-DED GRCop-42.

While nanoindentation provides valuable insights into localized mechanical properties, translating this data into predictions of tensile strength requires further refinement of existing models to account for the complexities of stress and strain interactions during indentation. Several studies have aimed to correlate the data obtained from indentation tests with conventional mechanical properties of the material, particularly tensile strength.^62,71^ Gao et al.^70^ developed an approach based on expanding cavity theory, incorporating the *H*_*ind*_*/σ*_*y*_ and *E/σ*_*y*_. Based on this model, Eq. 10 was proposed to account for the work-hardening exponent and predict the YS as follows:

10 $$\frac{{H}_{ind}}{{\sigma }_{y}}=\frac{2}{3}\left[\left(1-\frac{1}{n}\right)+(\frac{3}{4}+\frac{1}{n}){(\frac{1}{3}\frac{E}{{\sigma }_{y}}\text{tan}\alpha )}^{n}\right]$$

where *E* is elastic modulus and *α* is the Berkovich indenter effective cone angle, which is taken to be 19.7°.^62,72^ The calculated values of the initial YS by nano-indentation test results of each of the samples in this study, including L-PBF GRCop-42 and GRCop-84, as well as LP-DED GRCop-42, are provided in Fig. 9. For comparison, actual YS obtained by the tensile test are provided side by side with the values of YS derived from nanoindentation. The model proposed by Gao et al.^70^ provides a reasonable approximation for predicting the YS of L-PBF GRCop-42 and GRCop-84, but not for the LP-DED GRCop-42 samples. While the predicted values for L-PBF GRCop-42 and GRCop-84 closely align with those obtained from uniaxial tensile tests (with differences ranging from 2% to 16%), the discrepancy for LP-DED GRCop-42 is substantially larger, reaching up to 55%.

The differential accuracy of the Gao et al. model in predicting YS between L-PBF and LP-DED GRCop-42 can be primarily linked to the microstructural anisotropy introduced by the respective AM techniques. L-PBF produces a more isotropic microstructure with smaller, randomly oriented grains, creating a uniform mechanical response that fits well within the assumptions of models based on average bulk properties.

Conversely, LP-DED inherently promotes a strong crystallographic texture due to its inherent thermal gradients aligned with specific crystallographic directions. Such a pronounced texture induces directional mechanical properties that vary substantially depending on the loading orientation relative to grain alignment. This anisotropy complicates the mechanical response beyond the scope of models like Gao et al.’s, which generally assume isotropic or weakly textured material behavior. Moreover, the grain size disparity, where LP-DED features significantly coarser grains compared to L-PBF, also influences deformation mechanisms, such as grain boundary-strengthening effects. Larger grains reduce the density of grain boundaries, effectively lowering yield strength. Since the Gao model does not explicitly incorporate grain size effects or texture-induced anisotropy, its predictive capability diminishes for LP-DED samples.

Additionally, the thermal history differences between L-PBF and LP-DED alter residual stress distributions and phase stability, further affecting mechanical properties in a manner not accounted for by the Gao model. Therefore, the greater discrepancy observed in LP-DED samples highlights the necessity of incorporating texture-aware modeling strategies that account for anisotropic plasticity and microstructural heterogeneity. Such approaches, potentially coupled with experimental characterization like EBSD texture quantification, could enhance YS predictions for strongly textured AM alloys beyond what Gao et al.'s isotropic assumptions allow. These discrepancies provide valuable insight for further research aimed at quantitatively integrating microstructural features, such as texture, precipitation, and grain boundary characteristics, into predictive models, thereby improving their accuracy and reliability for diverse AM techniques.

### Indentation-Related Quantitative Parameters

Although the primary objective of this study is to evaluate the correlation between small-scale mechanical properties (obtained via nanoindentation) and macroscale tensile properties, additional valuable insights can be extracted from the load–depth curves generated during nanoindentation testing. For this reason, a dedicated section of the manuscript focuses on the quantitative parameters derived from these curves. Analyzing the load–depth response provides meaningful metrics that capture key aspects of the material's deformation behavior, including energy dissipation, plasticity index, and elastic recovery ratio. These parameters offer a deeper understanding of the mechanical behavior of the material beyond simple hardness measurements. In nanoindentation analysis, the hardness-to-reduced modulus ratio ($\frac{H}{{E}_{r}}$) is commonly examined as an indicator of material performance. While it is often associated with wear resistance in general contexts, in this study, it is used primarily to characterize the elastic–plastic response and assess deformation behavior at small scales. Another significant metric, $\frac{{H}^{3}}{{E}_{r}^{2}}$, often referred to as the yield pressure, measures the material's resistance to plastic deformation under load.^73^ A higher yield pressure indicates a stronger resistance to permanent deformation, which is especially important for materials used in load-bearing or high-stress applications. Such metrics allow for the characterization of the material under various loading conditions, aiding in the design and selection of materials for specific engineering applications.

**Fig. 10:**
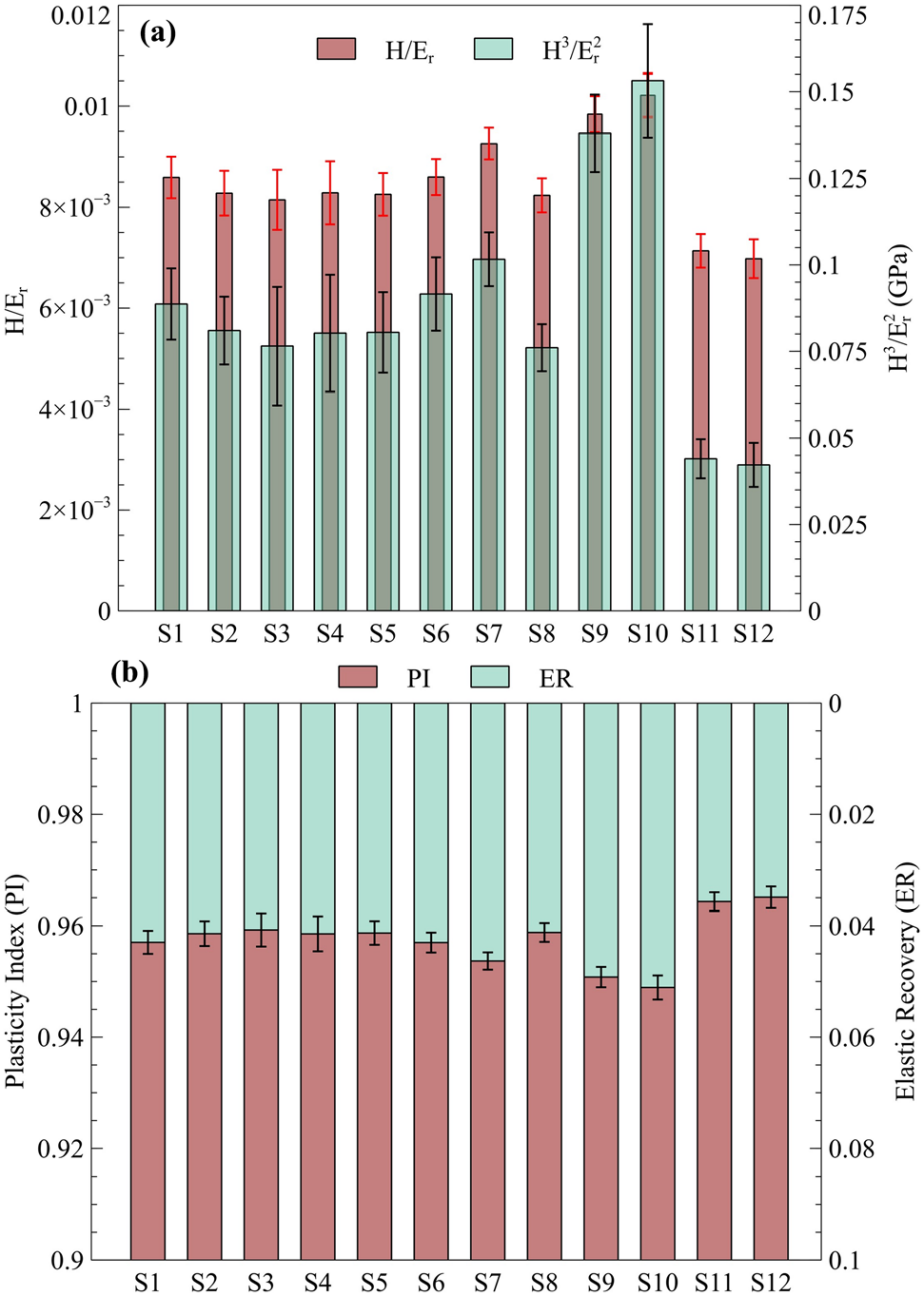
(a) Wear resistance index, $\frac{H}{{E}_{r}}$ and $\frac{{H}^{3}}{{{E}_{r}}^{2}}$, and (b) plasticity index (*PI*) and elastic recovery (*ER*) for GRCop samples: *S1–S8* represent L-PBF GRCop-42, *S9* and *S10* represent L-PBF GRCop-84, and *S11* and *S12* represent LP-DED GRCop-42.

Figure 10a presents the comparison of $\frac{H}{{E}_{r}}$ and $\frac{{H}^{3}}{{E}_{r}^{2}}$ values for the studied materials, highlighting key differences in wear resistance and plastic deformation resistance across various manufacturing techniques and material compositions. The data reveal that the L-PBF GRCop-84 exhibits a notably higher wear resistance index compared to the L-PBF GRCop-42. Furthermore, when comparing GRCop-42 fabricated via two manufacturing methods, L-PBF and LP-DED, the L-PBF sample demonstrated significantly higher $\frac{H}{{E}_{r}}$ and $\frac{{H}^{3}}{{E}_{r}^{2}}$ values, indicating superior resistance to plastic deformation and wear. This enhanced performance is attributed to the finer microstructure produced by the L-PBF process, which contributes to improved strength and mechanical stability under applied loads. It is important to emphasize that the indices $\frac{H}{{E}_{r}}$ and $\frac{{H}^{3}}{{E}_{r}^{2}}$ primarily represent a material's theoretical wear response and wear-resistant capacity rather than providing direct measurements of wear characteristics, such as wear volume or weight loss. There is a strong correlation between material hardness and wear resistance, with harder materials generally exhibiting greater resistance to wear.^74,75^ This principle helps to contextualize the observed trends, as the increased hardness associated with the L-PBF process contributes to the superior wear performance of these materials.

In addition to these metrics, the total work of the indenter during the loading phase (U_t_) consists of two parts: elastic energy (U_e_) and plastic energy (U_p_), with U_t_ = U_e_ + U_p_. The elastic energy represents the recoverable portion released during unloading, reflecting the material's elastic recovery. The plastic energy, in contrast, corresponds to the permanent deformation caused by the indentation. The total indentation work can be graphically interpreted as the area enclosed under the loading curve and the displacement axis, representing the energy involved in the deformation process (see Fig. 7a). In contrast, the elastic energy is represented by the area under the unloading curve and the displacement axis, reflecting the recoverable portion of the work. Two critical metrics derived from these energy components are the elastic recovery ratio (U_e_/U_t_) and the plasticity index (U_p_/U_t_).

When these energy ratios are correlated with the hardness-to-reduced modulus ratio ($\frac{H}{{E}_{r}}$), they yield valuable information about the material’s mechanical response. For instance, the relationship between the elastic recovery ratio and $\frac{H}{{E}_{r}}$ can be expressed as:

11 $$\text{Elastic recovery}=\frac{{U}_{e}}{{U}_{t}}\approx 5(\frac{{H}_{r}}{{E}_{r}})$$

12 $$\text{Plasticity index}=\frac{{U}_{P}}{{U}_{t}}\approx 1-5(\frac{{H}_{r}}{{E}_{r}})$$

Such correlations highlight the material's capacity to withstand both elastic and plastic deformation. Figure 10b illustrates another micromechanical behavior by presenting the plasticity index and elastic recovery values for the materials. While the differences in these indices are relatively marginal across the materials, notable trends can still be observed. L-PBF GRCop-84 exhibits a slightly lower plasticity index compared to both L-PBF GRCop-42 and LP-DED GRCop-42, indicating that L-PBF GRCop-84 has a reduced tendency for permanent deformation under load. Conversely, LP-DED GRCop-42 shows a higher plasticity index compared to its L-PBF counterpart, which implies that the LP-DED variant is more prone to plastic deformation. In terms of elastic recovery, which represents the ability of a material to return to its original shape after deformation, L-PBF GRCop-84 demonstrates the highest value among these materials. This reflects its greater capacity for reversible deformation, contributing to its overall resilience. On the other hand, LP-DED GRCop-42 exhibits the lowest elastic recovery, highlighting its reduced ability to recover elastically, which could limit its performance in applications requiring repeated loading and unloading cycles.

### Limitations and Future Studies

While the objective of this study was to establish methods for correlating bulk-scale (tensile) and nanoscale (hardness) mechanical properties of the GRCop family of alloys, several limitations remain and may serve as promising directions for future research.

Classical models, such as those proposed by Tabor,^61^ while useful for correlating bulk and small-scale mechanical properties, rely on the assumption of microstructural homogeneity. This simplification limits their accuracy when applied to AM materials, which often exhibit significant microstructural heterogeneity, anisotropy, and process-induced defects. In particular, grain orientation, porosity, and residual stresses, features intrinsic to AM, are not considered in these models. Furthermore, this study did not systematically examine the influence of factors like build height, spatial location on the build plate, or subtle variations in process parameters, all of which can affect thermal gradients and solidification behavior, thereby altering the mechanical response. Incorporating microstructural variability and directional dependence into strength prediction models remains a critical direction for future research in the AM field.

Another limitation lies in the reliance on tensile parameters such as Young’s modulus and the work-hardening exponent when using models like Gao’s^71^ to predict material strength. Although these parameters can be estimated from nanoindentation, such estimates may introduce additional uncertainty. Developing more advanced models that eliminate the need for such input parameters would offer a more robust and autonomous method for predicting strength solely from indentation data, reducing dependence on destructive testing.

Finally, though nanoindentation is a fast, convenient, and semi-destructive tool for data collection, the results are sensitive to test parameters such as indentation size and type, loading rate, and unloading procedures. Systematically considering these factors in experimental design can improve the reliability of correlating nanoindentation measurements with bulk tensile properties. Given the speed and cost-effectiveness of nanoindentation, generating large datasets under varied conditions provides a valuable opportunity for researchers to establish stronger predictive relationships. When combined with statistical or machine learning frameworks, this approach can support more accurate and scalable property estimations in complex AM materials.

## Conclusions

This study has investigated the mechanical and microstructural properties of additively manufactured GRCop-42 and GRCop-84 alloys, focusing on correlating nanoindentation metrics with tensile properties to determine the hardness-strength relationship. Based on the findings and observations from the experimental results, the following conclusions can be drawn:

- L-PBF produces a fine microstructure with moderate texture. In contrast, LP-DED generates elongated, columnar grains with strong texture aligned along the build direction.
- Tensile testing showed that L-PBF GRCop-42 exhibited higher strength but lower stiffness than LP-DED GRCop-42. In addition, L-PBF GRCop-84 achieved the highest strength, compared to L-PBF GRCop-42.
- The indentation load–depth results reveal that L-PBF GRCop-84 exhibits the highest hardness, while LP-DED GRCop-42 shows the greatest depth, indicating the lowest hardness among the tested samples. L-PBF GRCop-42 falls in between, reflecting moderate hardness.
- The correlation factors for UTS vary significantly across alloys and manufacturing methods, influenced by microstructural features like crystallographic texture and grain size. L-PBF GRCop-42 exhibits a consistent range (2.93–3.25), aligning with established models, while L-PBF GRCop-84 and LP-DED GRCop-42 show lower values (2.91–2.99) due to processing differences.
- The resulting microstructural analysis and mechanical testing reveal that the L-PBF GRCop-42 samples, characterized by finer and more homogeneous grain structure along with moderate crystallographic texture, exhibit enhanced mechanical performance compared to their LP-DED counterparts. Moreover, this uniformity in microstructure facilitates a stronger correlation between small-scale properties, such as hardness, and bulk-scale mechanical behavior, thereby enhancing the reliability of property prediction across scales.
- Future work should incorporate detailed microstructural features and spatial variability to better capture the anisotropy and heterogeneity of AM materials, potentially through data-driven modeling approaches.
